# Rhein from a traditional herbal formula attenuates hepatic fibrosis via ALDH1A3-mediated retinoic acid metabolism

**DOI:** 10.1186/s13020-026-01465-2

**Published:** 2026-07-14

**Authors:** Kai Yu, Minqi Chen, Wanchao Hou, Jinhua Lu, Qianhan Liu, Wanrong Zeng, Yan Xie, Zhengcai Du, Xiaotao Hou, Erwei Hao, Jiagang Deng

**Affiliations:** 1 Guangxi Key Laboratory of Efficacy Study on Chinese Materia Medica, Guangxi University of Chinese Medicine, No. 13 Wuhe Avenue, Qingxiu District, Nanning, 530200 China; 2 Guangxi Key Laboratory of TCM Formulas Theory and Transformation for Damp Diseases, Guangxi University of Chinese Medicine, Nanning, 530200 China; 3 Guangxi Collaborative Innovation Center of Study on Functional Ingredients of Agricultural Residues, Guangxi University of Chinese Medicine, Nanning, 530200 China; 4 University Engineering Research Center of Reutilization of Traditional Chinese Medicine Resourcs, Nanning, 530200 China; 5 College of Pharmacy, Guangxi University of Chinese Medicine, Nanning, 530200 China

**Keywords:** Liver fibrosis, Hepatic stellate cell, Rhein, ALDH1A3, Extracellular matrix, Retinoic acid metabolism, Multi-omics

## Abstract

**Background:**

Hepatic fibrosis is a prevalent outcome of chronic liver diseases. Although activation of hepatic stellate cell (HSC) is a primary driver of fibrogenesis, therapeutic strategies targeting retinoic acid (RA) metabolism in HSCs remain insufficiently investigated. This study aimed to elucidate the material basis of Huanggen formula (FHG), identify its principal bioactive constituents, and clarify the underlying antifibrotic mechanisms.

**Method:**

The antifibrotic efficacy of FHG was assessed in a CCl_4_-induced mouse model. Widely-targeted and untargeted metabolomics were conducted to characterize FHG constituents and serum-absorbed components, followed by high-content screening to evaluate anti-HSC activation activity. Rhein was further examined in TGF-β1-stimulated LX-2 cells and CCl_4_-induced mice. Label-free proteomics, multiple reaction monitoring quantification, DARTS, CETSA, and molecular docking were performed to identify the molecular targets of rhein. ALDH1A3 inhibition, knockdown, and overexpression were employed to verify its functional role in LX-2 cells.

**Results:**

FHG markedly alleviated metabolic disturbances and histopathological injury in fibrotic mice. Metabolomic analysis identified anthraquinones and flavonoids as the principal active classes, with rhein exhibiting potent anti-HSC activation effects. Rhein suppressed LX-2 activation, extracellular matrix (ECM) accumulation, and migration, and mitigated CCl_4_-induced hepatic fibrosis. Proteomic analysis indicated that rhein regulated ECM remodeling, mitochondrial function, and RA metabolism, accompanied by significant upregulation of DHRS4 and ALDH1A3. Mechanistically, rhein directly targeted to ALDH1A3, enhanced its expression, and increased intracellular ATRA level. The inhibition of ALDH1A3 partially abrogated the effects of rhein. Knockdown and overexpression experiments verify that ALDH1A3 is a critical negative regulator of HSC activation.

**Conclusion:**

Rhein is a key antifibrotic constituent of FHG and suppresses hepatic fibrosis by targeting ALDH1A3 and restoring RA metabolism. The ALDH1A3-ATRA axis represents a central mechanism and a promising therapeutic target.

**Graphical Abstract:**

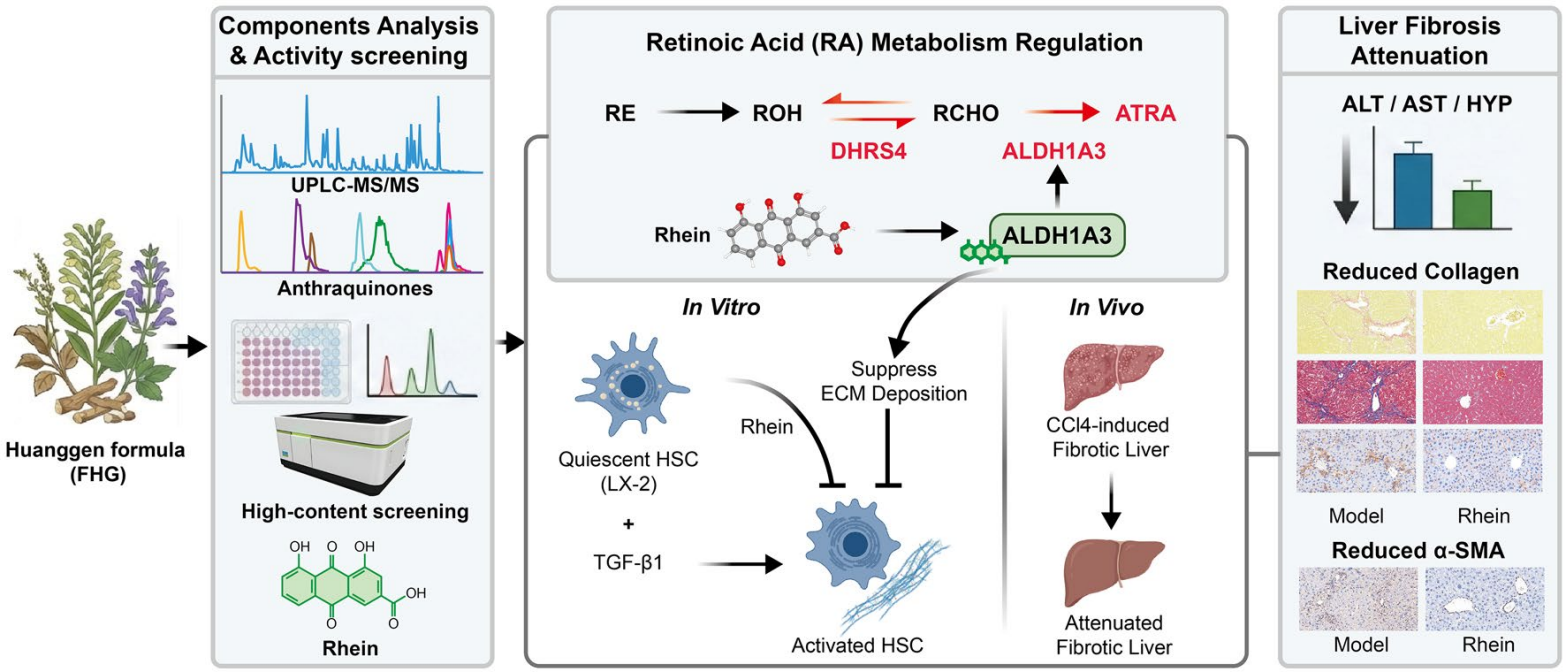

**Supplementary Information:**

The online version contains supplementary material available at 10.1186/s13020-026-01465-2.

## Highlights

1. Herb and serum metabolomics defined FHG’s chemical basis, highlighting anthraquinones.
2. High-content screening identified rhein as the principal antifibrotic compound.
3. Rhein suppressed HSC activation by through modulation of RA metabolism via ALDH1A3.
4. Rhein upregulated ALDH1A3 and elevated ATRA level to inhibit HSC activation.
5. The ALDH1A3-ATRA axis is critical for regulation of HSC activation.

**Supplementary Information:**

The online version contains supplementary material available at 10.1186/s13020-026-01465-2.

## Introduction

Hepatic fibrosis represents a conserved yet pathological wound-healing response to chronic liver injury, including viral hepatitis, alcohol abuse, and metabolic dysfunction-associated steatotic liver disease (MASLD). This dynamic process is defined by the excessive extracellular matrix (ECM) accumulation, primarily resulting from by the transdifferentiation of quiescent hepatic stellate cells (HSCs) into proliferative, myofibroblast-like cells. This phenotypic transition is regulated by a complex intracellular signaling network, in which transforming growth factor-β (TGF-β) serves as a predominant mediator. Through Smad-dependent transcriptional programs and additional profibrotic mediators such as VEGF and PDGF, activated HSCs synthesize substantial amounts of collagen and fibronectin (FN) [1], while concurrently inhibiting ECM degradation [2]. Although the canonical TGF-β/Smad signaling pathway remains fundamental to fibrogenesis, accumulating evidence indicates that HSC activation is not exclusively signaling-driven but also metabolically coordinated. Recent studies identify mitochondrial metabolic reprogramming as a prerequisite for HSC fate transition, conferring the bioenergetic and redox adaptability necessary for myofibroblastic differentiation [3, 4]. Therefore, pharmacological approaches designed to attenuate HSC activation or reestablish a quiescent phenotype have attracted considerable interest, and several small-molecule inhibitors have demonstrated antifibrotic efficacy in preclinical models [5, 6]. Collectively, these findings support the therapeutic potential of targeting key molecular and metabolic regulators that govern HSC activation.

A defining metabolic characteristic of HSC quiescence is the extensive intracellular storage of vitamin A (retinoids). During fibrotic progression, these retinoid reserves are rapidly depleted, constituting a metabolic “tipping point” that promotes HSC activation [7]. Bioactive retinoids, particularly all-trans retinoic acid (ATRA), exert potent regulatory effects on mitochondrial function and ECM homeostasis, thereby modulating HSC activation [8]. However, the context-dependent effects of retinoic acid (RA) in fibrosis complicate its therapeutic application, and the upstream biosynthetic mechanisms controlling RA production during HSC activation remain insufficiently defined. Aldehyde dehydrogenase 1 family member A3 (ALDH1A3), a rate-limiting enzyme catalyzing the irreversible oxidation of retinaldehyde to RA, has recently emerged as an important regulator of cellular homeostasis. Beyond its role in retinoid metabolism, ALDH1A3 detoxifies reactive aldehydes generated during lipid peroxidation [9]. Impairment of ALDH1A3 activity induces lipid peroxidation, fatty acid dyshomeostasis, and hepatic inflammatory and fibrotic cascades [10]. Consistently, suppression of ALDH1A3 promotes microvesicular steatosis and HSC fibrotic degeneration [11]. In addition to its canonical functions, ALDH1A3 modulates mitochondrial bioenergetics and tissue remodeling, and its expression is closely associated with TGF-β1 signaling [12, 13]. Although dysregulation of the ALDH family has been increasingly linked to fibrosis-related disorders, the specific contribution of ALDH1A3 to HSC activation and hepatic fibrosis remains largely undefined.

Traditional herbal formulas constitute a valuable resource for the identification of multi-target antifibrotic agents. Based on longstanding clinical practice in Guangxi, the Huanggen formula (FHG) was developed, comprising *Prismatomeris tetrandra* (Huanggen), *Isodon ternifolius* (Sanjiemei), *Phyllanthus urinaria* (Yexiazhu), *Gynostemma pentaphyllum* (Jiaogulan), *Astragalus mongholicus* (Huangqi), *Atractylodes macrocephala* (Baizhu), and *Panax notoginseng* (Sanqi) [14]. Clinical observations indicate that FHG improves liver function and mitigates fibrotic progression in chronic hepatitis, and our previous studies have experimentally validated its hepatoprotective effects [15]. These findings position FHG as a clinically grounded formula with substantial antifibrotic potential and provide a rationale for investigating its active constituents. Rhein, a major anthraquinone derivative derived from FHG components (e.g., *Rheum* species), has been widely recognized for its antifibrotic properties. It suppresses HSC activation and ECM deposition through modulation of signaling pathways, including TGF-β/Smad, NF-κB, MAPK, and PI3K/Akt [16, 17]. However, the primary molecular targets of rhein remain undefined, and whether rhein regulates retinol metabolism has yet to be determined.

In this study, metabolomics and high-content screening (HCS) were integrated to systematically characterize the antifibrotic constituents of FHG. Rhein was identified as a principal active constituent that directly targets ALDH1A3. By restoring the rhein-ALDH1A3-ATRA axis, rhein attenuates ECM deposition in activated HSCs. These findings provide novel mechanistic insight into metabolism-oriented therapeutic strategies for hepatic fibrosis.

## Materials and methods

### Chemicals and reagents

FHG was provided by the Pharmaceutical Factory of Guangxi University of Chinese Medicine. The natural active compounds were purchased from Push Bio-Technology (Chengdu, China) (Table S1). DMEM, fetal bovine serum (FBS), 1% penicillin–streptomycin (P/S), and 0.25% trypsin–EDTA were obtained from Gibco (Carlsbad, CA, USA). ATRA, ALDH1A3 inhibitor (MCI-INI-3), carbon tetrachloride (CCl_4_), and pirfenidone (PFD) were also purchased from MedChemExpress (Shanghai, China). Primary antibodies against FN, α-SMA, COL1A1, ALDH1A3, DHRS4, and GAPDH, as well as HRP- or CoraLite^®^ Plus 488/594-conjugated secondary antibodies (Table S2), were purchased from ProteinTech (Wuhan, China). ALT and AST activity assay kits and the HYP content assay kit were obtained from Jiancheng (Nanjing, China); Lipofectamine 3000 was purchased from Invitrogen (Carlsbad, CA, USA); recombinant human ALDH1A3 protein was purchased from CUSABIO (Wuhan, China); siRNAs were synthesized by Sangon (Shanghai, China); pCDH-puro 3Xflag-ALDH1A3 was offered by SYNBIO (Suzhou, China); the BCA protein assay kit was obtained from Pierce (Waltham, MA, USA); cell total RNA isolation kit, RT SuperMix, and SYBR qPCR kit were purchased from Vazyme (Nanjing, China).

### Preparation of FHG drug-containing serum

SPF male SD rats (300–320 g, n = 12) were obtained from Hunan SJA Laboratory Animal Co., Ltd. After a 1-week acclimatization period, the rats were randomly assigned to a blank serum group and an FHG group (n = 6 per group). Rats in the FHG group received FHG extract by oral gavage at a daily dose of 3.5 g/kg, whereas the blank group was administered an equivalent volume of normal saline. The detailed procedure for preparation and collection of FHG-containing serum was performed as previously described by Pan et al. [18].

### Animal experiments

Male C57BL/6J mice (SPF, 6–8 weeks old, 18–22 g, n = 108) were obtained from Hunan SJA Laboratory Animal Co., Ltd. After 1 week of acclimatization, the mice were randomly allocated into nine groups (n = 12 per group): normal control (NC), model (CCl_4_), PFD, FHG low-dose (FHG_L), FHG medium-dose (FHG_M), FHG high-dose (FHG_H), rhein low-dose (RheL), rhein medium-dose (RheM), and rhein high-dose (RheH). Hepatic fibrosis was induced by intraperitoneal injection of CCl_4_ (diluted 1:4 in corn oil) twice weekly for 8 weeks. The NC group received corn oil alone. Beginning at week 4, mice were administered vehicle, PFD (200 mg/kg), FHG (1.25, 2.5, or 5 g/kg), or rhein (25, 50, or 100 mg/kg) [19] by oral gavage every other day until the end of the experiment. Rhein was dissolved in 0.5% carboxymethylcellulose sodium. After a 24 h fast, mice were euthanized, and serum and liver tissues were harvested for subsequent analysis. All animal procedures were approved by the Ethics Committee of Guangxi University of Chinese Medicine (GXTCMU-EC20250805-167).

### Widely targeted metabolomics analysis of drug components

Drug samples were extracted and analyzed by liquid chromatography-tandem mass spectrometry (LC–MS/MS) according to previously described methods [20, 21]. Briefly, FHG and herb powder were extracted with 70% methanol, vortexed, centrifuged, and filtered. After vortexing and overnight precipitation at 4 °C, the supernatant was centrifuged and filtered before UPLC-ESI-QTRAP analysis. Chromatographic separation was conducted on an SB-C18 column using a formic acid–water/acetonitrile gradient. Detection was performed using an ESI-QTRAP system in both positive and negative ion modes under multiple reaction monitoring (MRM) conditions. Metabolites were qualitatively and quantitatively analyzed using MWDB and Analyst 1.6.3, with MultiaQuant applied for peak integration.

### Untargeted metabolomics analysis of FHG drug-containing serum

Serum samples were mixed with 20% acetonitrile–methanol (containing internal standards), centrifuged, and incubated for 30 min. The supernatant was evaporated under nitrogen, reconstituted in 70% methanol, and centrifuged again to obtain the supernatant for LC–MS/MS analysis. Chromatographic separation was performed on a Waters ACQUITY Premier HSS T3 column (1.8 µm, 2.1 × 100 mm) maintained at 40 °C. The mobile phase comprised 0.1% formic acid in water and acetonitrile, delivered with a linear gradient (5–99% B) at a flow rate of 0.35 mL/min. Mass spectrometry detection was carried out on a Q Exactive HF-X mass spectrometer using electrospray ionization in positive (3.5 kV) and negative (3.2 kV) ion modes. Full-scan acquisition was conducted over an m/z range of 84–1,250 (resolution 35,000), followed by data-dependent data-dependent MS^n^ acquisition.

### HCS assay

LX-2 cells were seeded in black, clear-bottom 96-well plates and co-treated with 10 ng/mL TGF-β1 and 23 representative monomer compounds at gradient concentrations (0.02, 0.1, 0.5, 2, 10, and 50 μg/mL) for 48 h. After incubation, FN immunofluorescence staining was performed as previously described [22]. Following incubation with the CoraLite Plus 488-conjugated FN primary antibody, the cells were subjected to nuclear counterstaining. Images were captured using the Operetta CLS high-content imaging system (PerkinElmer, USA). Quantitative analysis of FN expression and cell number was conducted using Cellomics Cell Health Profiling software.

### Histological examination

Liver tissues were fixed in 4% paraformaldehyde, paraffin-embedded, and sectioned at 5 μm. Hematoxylin and eosin (H&E), Masson’s trichrome, and Sirius Red staining were performed according to standard protocols. Stained sections were examined under a light microscope. Collagen deposition was quantified using Image Pro Plus 6.0 software.

### Immunohistochemistry (IHC) staining

Fixed liver tissue sections were deparaffinized, rehydrated, and subjected to heat-induced antigen retrieval following quenching of endogenous peroxidase activity with 3% H_2_O_2_. After blocking with 10% goat serum, sections were incubated overnight at 4 °C with primary antibodies, followed by incubation with secondary antibodies. Immunoreactivity was visualized using DAB, and nuclei were counterstained with hematoxylin. Images were acquired by light microscopy and quantitatively analyzed using ImageJ software.

### Immunofluorescence (IF) assay

To assess the effects of rhein on ECM deposition, LX-2 cells were co-treated with 10 ng/mL TGF-β1 in the presence or absence of 5 or 10 μg/mL rhein for 48 h. For the ALDH1A3 inhibitor assay, LX-2 cells were co-treated with 10 ng/mL TGF-β1 in the presence or absence of 10 μg/mL rhein and 2 μM MCI-INI-3. For transient interference analysis of ALDH1A3, LX-2 cells were transfected with siRNA without drug treatment for 48 h. Treated cells were fixed in 4% paraformaldehyde, permeabilized with 0.2% Triton X-100, and blocked with 5% bovine serum albumin for 1 h at room temperature. Paraffin sections or cultured cells were incubated with primary antibodies against α-SMA, COL1A1, FN, and ALDH1A3, followed by incubation with corresponding secondary antibodies. Images were obtained using an Olympus BX53 microscope.

### Cell culture and treatment

Human hepatic stellate LX-2 cells were obtained from the Chinese Academy of Sciences Cell Bank (Shanghai, China). Cells were maintained in DMEM supplemented with 2% FBS and 1% P/S at 37 °C in a humidified incubator with 5% CO_2_ [23]. HSC activation was induced by TGF-β1 treatment. Rhein was administered at the indicated concentrations according to the experimental design.

### Cell viability assay

Cell viability was determined using the Cell Counting Kit-8 (CCK-8) assay. LX-2 cells were exposed to rhein at the indicated concentrations, followed by incubation with CCK-8 reagent. Absorbance was recorded at 450 nm, and the results were normalized to control values.

### qRT-PCR

Total RNA was isolated using the FastPure Cell/Tissue Total RNA Isolation Kit and reverse-transcribed into cDNA with HiScript II Q RT SuperMix for qPCR. Quantitative PCR was conducted using ChamQ SYBR Green Master Mix. Relative gene expression levels were calculated using the 2^⁻ΔΔCT^ method, with GAPDH serving as the internal control. Primers were listed in Table S3.

### Western blot (WB)

Total protein was separated by sodium dodecyl sulfate–polyacrylamide gel electrophoresis and transferred onto polyvinylidene fluoride membranes. After blocking with 5% goat serum, membranes were incubated with primary antibodies against FN, α-SMA, COL1A1, ALDH1A3, DHRS4, flag, and GAPDH, followed by incubation with HRP-conjugated secondary antibodies. Protein bands were detected using enhanced chemiluminescence Detection Reagent.

### Quantitative proteomic

LX-2 cells treated with rhein or vehicle were harvested and lysed for protein extraction. Equal amounts (100 μg) of protein were reduced, alkylated, and digested with trypsin as previously described [24]. The resulting peptides were analyzed by label-free quantification (LFQ) using a nanoLC system coupled to a Q Exactive HFX mass spectrometer (Thermo Fisher Scientific). Peptides were separated on a 50 cm analytical column with a 60 min linear gradient. MS data were acquired in data-dependent acquisition (DDA) mode. Raw data were processed using MaxQuant against the Swissprot human database. Differentially expressed proteins (DEPs) were identified according to predefined fold-change thresholds and adjusted *p*-values, followed by Gene Ontology (GO) and Kyoto Encyclopedia of Genes and Genomes (KEGG) pathway enrichment analyses.

### Drug affinity responsive target stability (DARTS)

DARTS assays were performed by incubating cultured cells or cell lysates with rhein, followed by limited proteolysis and WB analysis as previously describe [25].

### Molecular docking and ‌dynamical simulation

Molecular docking of rhein with ALDH1A3 was performed using AutoDock Vina. The binding conformation with the lowest binding energy was selected for molecular dynamics simulation using GROMACS to assess complex stability and key intermolecular interactions.

### MicroScale thermophoresis (MST) assay

MST assays were carried out using a Monolith NT.115 Pico instrument (NanoTemper, Germany). Briefly, recombinant human ALDH1A3 protein was labeled with the RED-NHS 2nd Generation kit, and labeling efficiency, protein adsorption, and aggregation were verified. A twofold serial dilution of the rhein ligand was then mixed with equal volumes of fluorescently labeled ALDH1A3. Samples were loaded into standard uncoated capillaries, and the dissociation constant (Kd) was measured in Binding Affinity mode using NanoTemper analysis software.

### MRM quantification of cellular ATRA

Intracellular ATRA was quantified using a MRM method. Briefly, LX-2 cells were lysed by ultrasonication, and proteins were precipitated with three volumes of methanol. The resulting supernatant was filtered and analyzed using a SHIMADZU LC-40 system coupled to a Triple Quad 3500 MS (AB SCIEX). Chromatographic separation was performed on an ACQUITY UPLC BEH C18 column (1.7 µm, 2.1 × 100 mm) at 40 °C with a methanol–water gradient. MS detection was conducted in negative ESI mode using the transition m/z 299.1 → 255.2 (CE: 24 V, DP: 106 V). Data were processed using Analyst 1.6.2, and ATRA concentrations were calculated from a standard curve (y = 0.001x−0.1009, R^2^ = 0.999) and normalized with total protein content.

### Cell transfection

siRNAs targeting ALDH1A3 and siNC were synthesized by Shenggong Biotech (Shanghai, China). LX-2 cells were transiently transfected with Lipofectamine 3000 according to the manufacturer’s instructions. At 24 h and 48 h post-transfection, knockdown efficiency was verified by qRT-PCR and WB, respectively, and transfection was visualized by fluorescence microscopy. The most effective siRNA sequence was subsequently cloned into a lentiviral vector to generate short hairpin RNA (shRNA) for stable ALDH1A3 silencing (SYNBIO Technologies, Suzhou, China). The siRNA and shRNA details were attached in Table S4. For overexpression experiments, full-length human ALDH1A3 cDNA was inserted into the pCDH-CMV-MCS-EF1-puro lentiviral vector (SYNBIO Technologies). Lentiviral particles were generated and used to infect LX-2 cells, followed by puromycin selection to establish stable ALDH1A3-overexpressing cell lines. Successful modulation of ALDH1A3 expression was confirmed before subsequent functional assays.

### Statistical analysis

Data are expressed as mean ± SD. Data distribution was evaluated for normality using the Shapiro–Wilk test prior to statistical comparison. For normally distributed datasets, intergroup differences were examined by one-way analysis of variance (ANOVA), followed by appropriate post hoc multiple-comparison tests. All statistical analyses were conducted using GraphPad Prism software. A *p* < 0.05 was considered statistically significant.

## Results

### FHG attenuates CCl_4_-induced liver fibrosis in mice

**Fig. 1:**
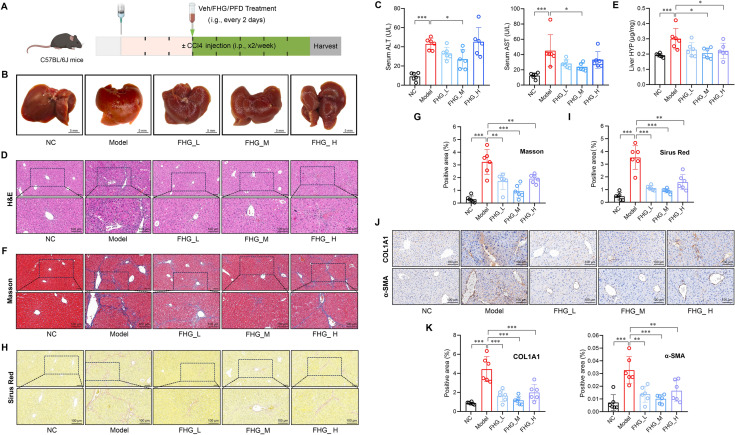
FHG attenuates CCl_4_-induced hepatic fibrosis in mice. **A** Schematic illustration of the experimental timeline for the CCl_4_-induced liver fibrosis model and the FHG treatment regimen. **B** Representative gross images of liver morphology. **C** Serum ALT and AST activities. **D** Representative micrographs of H&E-stained liver sections. **E** Quantitative determination of hepatic HYP content. **F**, **G** Masson’s trichrome staining of liver sections and corresponding quantitative morphometric assessment of collagen-positive areas (blue). **H**, **I** Sirius Red staining with quantitative evaluation of collagen fibers (red). **J** Representative IHC images of COL1A1 and α-SMA expression in liver tissues. **K** Quantitative analysis of the integrated optical density of COL1A1 and α-SMA protein expression. Data are presented as mean ± SD (n = 6). *p < 0.05, **p < 0.01, ***p < 0.001

To assess the therapeutic efficacy of FHG, fibrotic mice were treated with different doses of the formula (Fig. 1A). Gross examination revealed pale, nodular, and coarse liver surfaces in the model group, whereas FHG administration markedly improved hepatic coloration and alleviated surface irregularities, with the medium dose exhibiting the most pronounced effect (Fig. 1B). Consistently, FHG_M treatment significantly reduced the CCl_4_-induced elevation of serum ALT and AST levels, indicating attenuation of hepatocellular injury (Fig. 1C). Histopathological evaluation by H&E staining demonstrated that FHG markedly ameliorated architectural disruption and inflammatory cell infiltration (Fig. 1D). Meanwhile, HYP content, a representative biochemical indicator of collagen deposition, was significantly decreased in the FHG_M group (Fig. 1E). The anti-fibrotic effects were further corroborated by Masson’s trichrome and Sirius Red staining, which showed that FHG markedly suppressed excessive collagen accumulation in model mice, as supported by quantitative analysis (Fig. 1F–I). Moreover, IHC staining indicated that the elevated expression of COL1A1 and α-SMA in fibrotic livers was substantially reduced following FHG treatment (Fig. 1J, K). Collectively, these results indicate that FHG effectively alleviates ECM deposition and maintains hepatic structural integrity under chronic injury conditions.

### Compounds profiling and serum pharmacochemistry of FHG

**Fig. 2:**
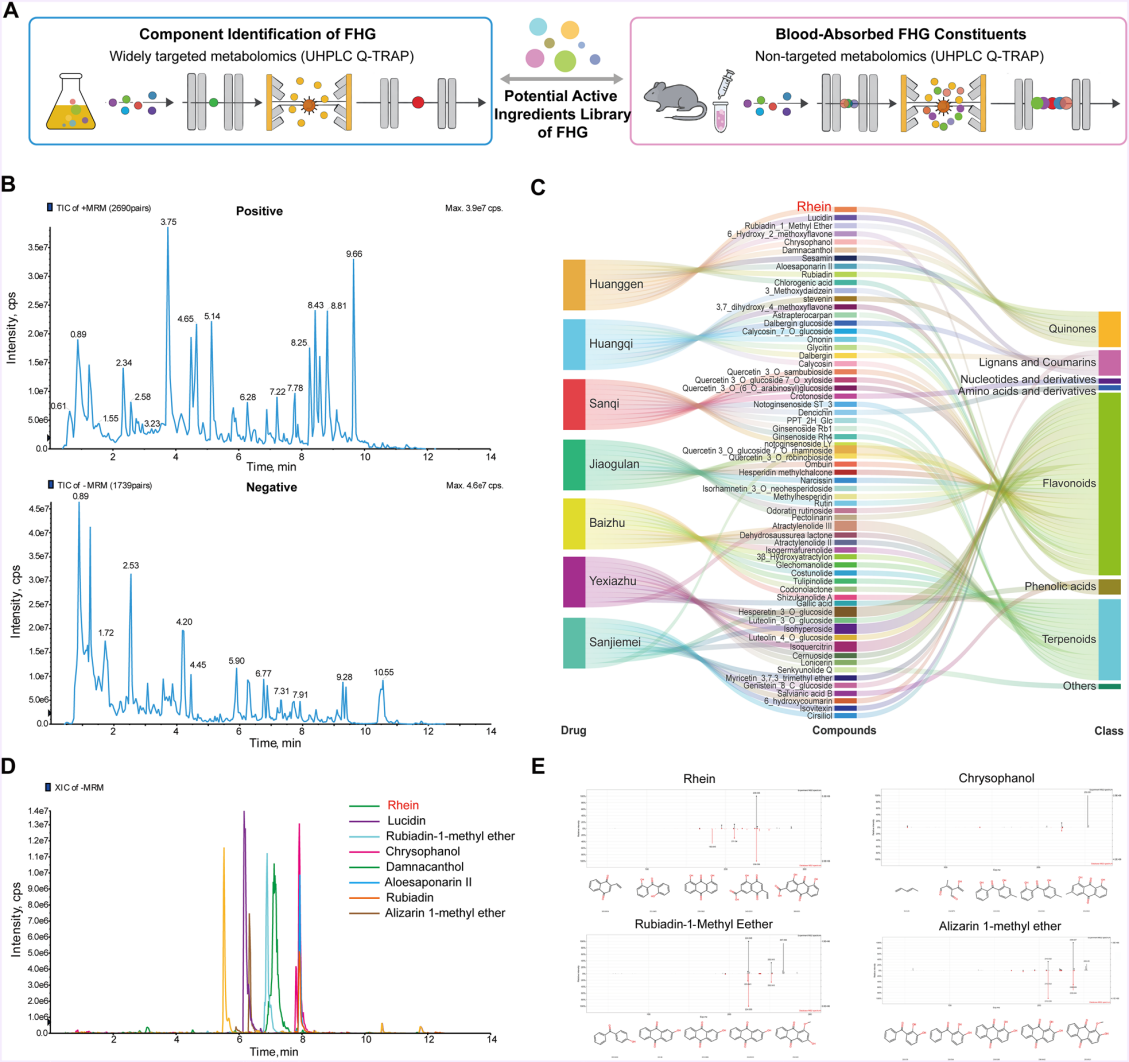
Compounds profiling and serum pharmacochemistry analysis of FHG. **A** Schematic workflow illustrating the strategy for elucidating the material basis of FHG. **B** Representative TIC of the FHG decoction acquired in positive and negative ESI modes, respectively. **C** Sankey diagram depicting the correlations among individual botanical ingredients, their principal constituents, and chemical classifications. **D** XIC of the major anthraquinones detected in the FHG decoction. **E** Representative MS2 spectral matching profiles of the principal anthraquinones in FHG-medicated serum

To systematically elucidate the material basis underlying the anti-fibrotic effects of FHG, widely targeted metabolomics was performed to characterize the chemical composition of the formula and its seven botanical constituents. Additionally, non-targeted metabolomics was conducted to identify bioactive components absorbed into the systemic circulation following FHG administration (Fig. 2A). A total of 263 annotated metabolites (MS intensity > 1 × 10^6^) were identified in the FHG decoction (Table S5), and representative total ion chromatograms (TIC) are shown in Fig. 2B. These constituents mainly comprise anthraquinones, flavonoids, terpenoids, and phenolic acids. Correlation analysis among individual herbs, predominant compounds, and chemical classes indicated that anthraquinones are mainly derived from Huanggen, whereas terpenoids predominantly originate from Baizhu. The remaining constituent herbs were enriched in flavonoids (Fig. 2C). Extracted ion chromatograms (XIC) identified eight major anthraquinones in the formula, including rhein, lucidin, rubiadin-1-methyl ether, chrysophanol, damnacanthol, aloesaponarin II, rubiadin, and alizarin 1-methyl ether (Fig. 2D). Notably, four of these anthraquinones (rhein, rubiadin-1-methyl ether, chrysophanol, and alizarin 1-methyl ether) demonstrated high-fidelity MS2 matching in the FHG-medicated serum (Fig. 2E). Based on the chemical characterization of FHG, a potential active ingredient library comprising 24 compounds (Table S1) was constructed for subsequent activity screening. The selection criteria included relative abundance in the formula and medicated serum, comprehensive database retrieval of pharmacological relevance, commercial availability, and cost-effectiveness.

### HCS identifies rhein as a key inhibitor of HSC activation

**Fig. 3:**
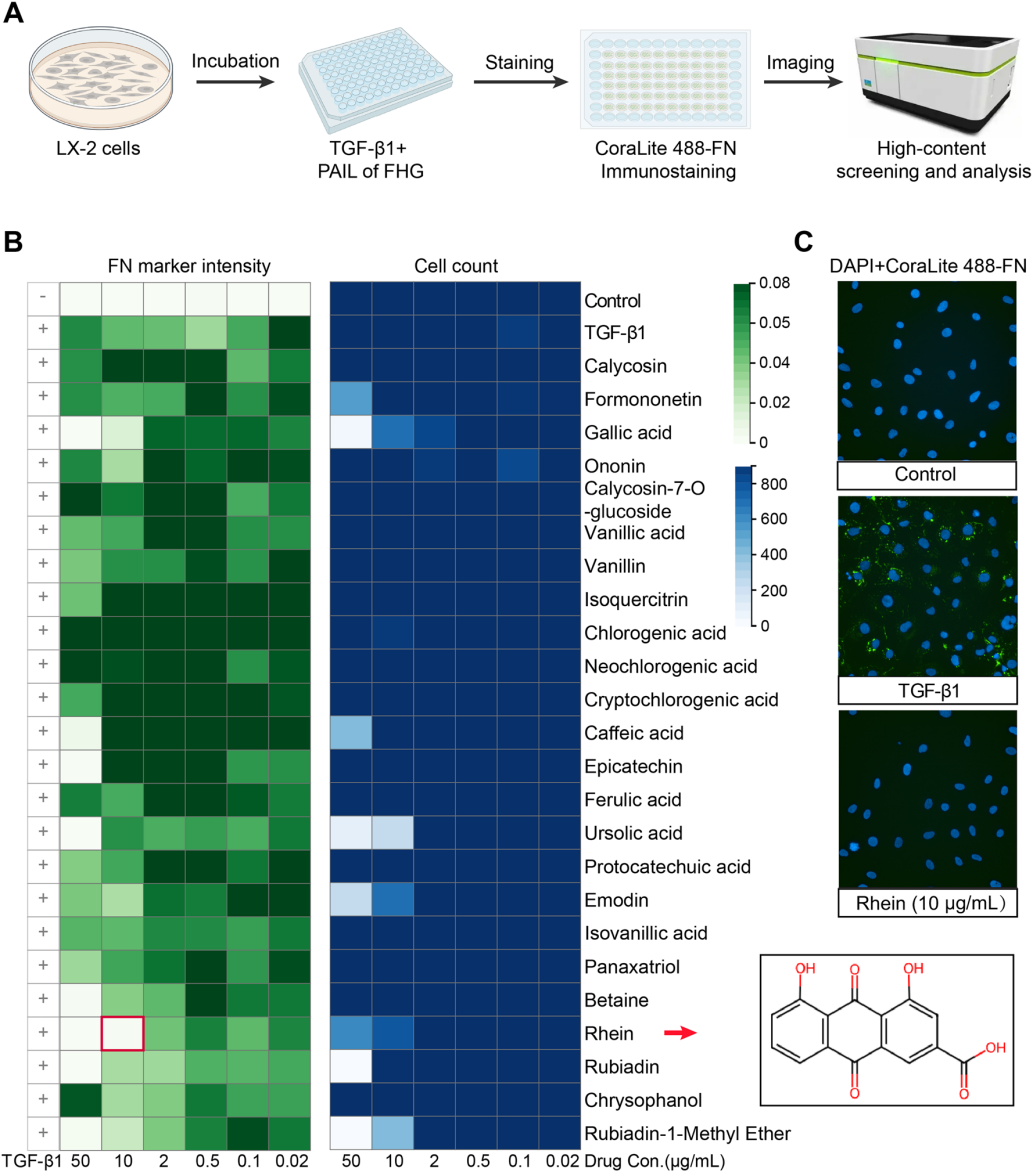
Identification of bioactive constituents in FHG via HCS. **A** Schematic workflow of the HCS assay. Created with BioRender.com. **B** Heatmap depicting FN fluorescence intensity (activation marker) and cell counts (viability marker) under various compound treatments. The chemical structure of rhein is presented in the lower panel. **C** Representative IF micrographs of LX-2 cells

To identify the principal anti-fibrotic constituents of FHG, an HCS platform was established using LX-2 cells, with FN expression and cell viability serving as dual readout parameters (Fig. 3A). Twenty-four representative compounds derived from FHG were assessed across six serial concentrations. Although several candidates, including rubiadin, gallic acid, and ursolic acid, markedly reduced FN levels, they concurrently induced significant cytotoxicity, thereby limiting their therapeutic applicability. Notably, rhein emerged as the most promising candidate, demonstrating potent FN-suppressive activity with minimal effects on cell viability. Specifically, rhein significantly decreased FN fluorescence intensity at concentrations as low as 2 μg/mL (Fig. 3B). Representative IF images further confirmed that treatment with 10 μg/mL rhein markedly attenuated FN accumulation in TGF-β1-activated LX-2 cells while preserving normal cellular morphology (Fig. 3C). Based on its superior efficacy and favorable safety profile, rhein was selected as the key bioactive constituent for subsequent mechanistic investigation.

### Rhein suppresses TGF-β1-induced activation of LX-2 cells

**Fig. 4:**
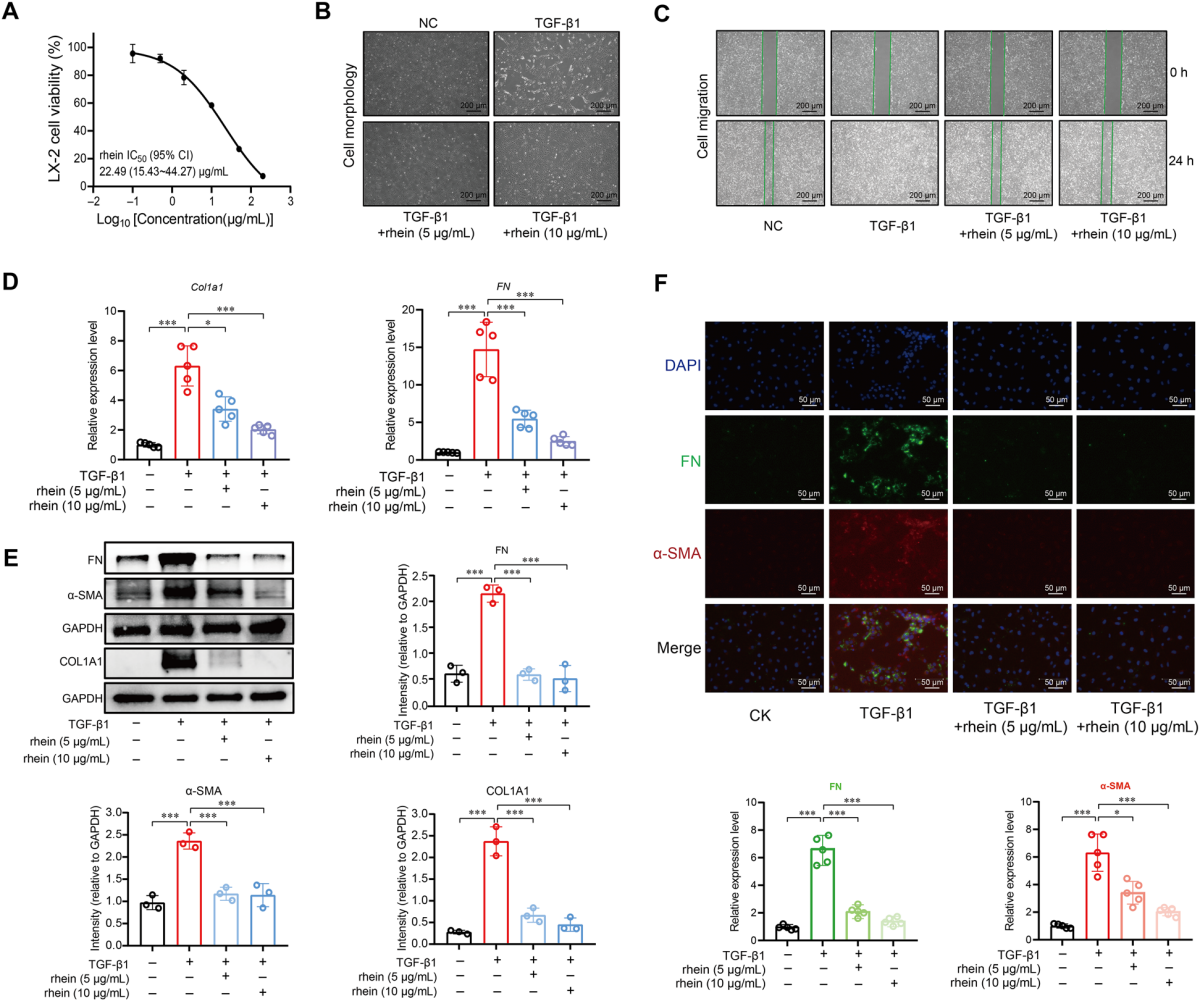
Rhein suppresses TGF-β1-induced activation of LX-2 cells in vitro. **A** Cell viability of LX-2 cells treated with various concentrations of rhein for 24 h determined by CCK-8 assay. **B** Representative images of cellular morphology under the indicated treatments. **C** Representative images of the cell migration assay under indicated treatments. **D** mRNA expression levels of *Col1a1* and *FN* in LX-2 cells, as measured by qRT-PCR. **E** Representative WB images and densitometric quantification of FN, α-SMA, and COL1A1 protein expression, with GAPDH serving as the loading control. **F** Representative IF images and quantitative analysis of FN (green) and α-SMA (red) in LX-2 cells; nuclei were counterstained with DAPI (blue). Data are presented as mean ± SD. * *p* < 0.05, ***p < 0.001

The cytotoxic effects of rhein on LX-2 cells were assessed using CCK-8 assays, yielding an IC_50_ of 22.49 μg/mL (95% CI 15.43–44.27 μg/mL) (Fig. 4A). Therefore, subtoxic concentrations (5 and 10 μg/mL) were selected for subsequent experiments. Morphologically, TGF-β1 stimulation induced a typical myofibroblastic transition characterized by an elongated fusiform morphology and cellular aggregation. In contrast, rhein treatment markedly reversed these alterations, restoring a more quiescent-like phenotype (Fig. 4B). In the wound-healing assay, rhein significantly attenuated TGF-β1-induced cell migration at both 5 and 10 μg/mL (Fig. 4C). At the molecular level, qRT-PCR analysis demonstrated that rhein significantly downregulated the mRNA expression of *COL1A1* and *FN* (Fig. 4D). Consistently, WB analysis showed reduced protein expression of FN, α-SMA, and COL1A1 following rhein treatment (Fig. 4E). These findings were further supported by immunofluorescence staining, which revealed a marked decrease in α-SMA and FN fluorescence intensity in rhein-treated groups (Fig. 4F). Collectively, these results indicate that rhein effectively inhibits TGF-β1-induced myofibroblastic activation and ECM production in LX-2 cells.

### Rhein attenuates CCl_4_-induced liver fibrosis in mice

**Fig. 5:**
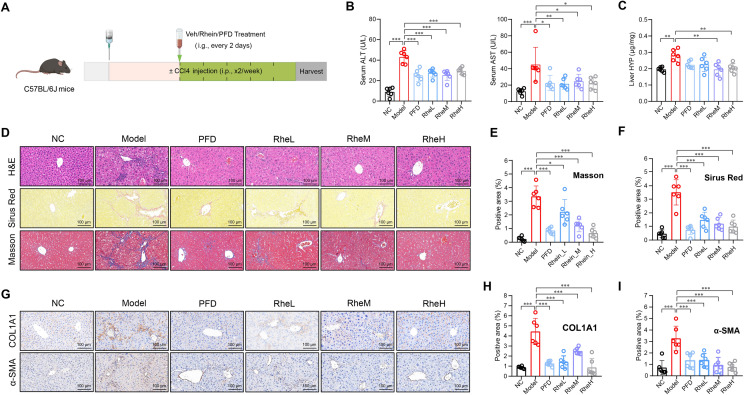
Rhein alleviates CCl_4_-induced hepatic injury and fibrotic deposition in mice. **A** Schematic illustration of the experimental design for CCl_4_-induced liver fibrosis and rhein, PFD treatment. **B** Serum activities of ALT and AST activities. **C** Hepatic HYP content. **D** Representative images of H&E, Sirius Red, and Masson’s trichrome staining in liver sections. **E**, **F** Quantitative morphometric assessment of Sirius Red-positive (red) and Masson-positive (blue) fibrotic areas. **G** Representative IHC images of COL1A1 and α-SMA expression in liver tissues. **H**, **I** Quantitative analysis of the positive staining areas for COL1A1 and α-SMA. Data are presented as mean ± SD (n = 6). *p < 0.05, **p < 0.01, ***p < 0.001

To validate the anti-fibrotic efficacy of rhein in vivo, a CCl_4_-induced liver fibrosis model was established (Fig. 5A). Rhein administration significantly reduced the CCl_4_-mediated elevation of serum ALT and AST levels, with the most pronounced decreases observed in the medium- and high-dose groups (Fig. 5B). In accordance with the improved biochemical parameters, hepatic HYP content was also markedly decreased following rhein treatment (Fig. 5C). H&E staining demonstrated that rhein mitigated CCl_4_-induced hepatic architectural disruption and inflammatory cell infiltration (Fig. 5D). Moreover, Sirius Red and Masson’s trichrome staining indicated that rhein markedly suppressed extensive collagen deposition, particularly in the medium- and high-dose groups (Fig. 5D–F). IHC analysis further confirmed that rhein significantly downregulated the expression of COL1A1 and α-SMA (Fig. 5G–I). Collectively, these findings indicate that rhein alleviates CCl_4_-induced liver injury and fibrotic deposition.

### Rhein induces distinct alterations in the LX-2 proteomic landscape

**Fig. 6:**
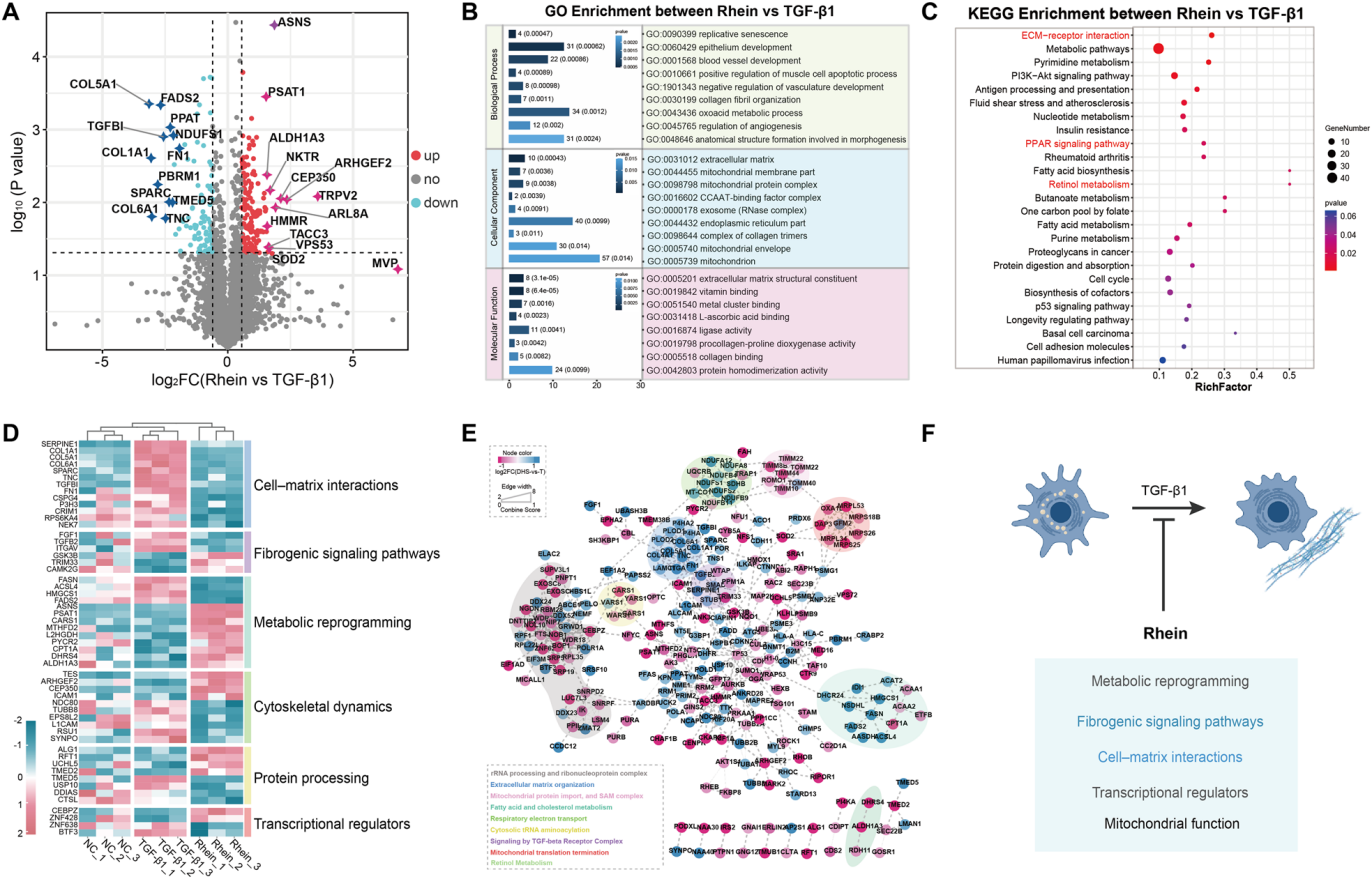
Quantitative proteomics reveals the impact of rhein on the proteomic landscape of activated LX-2 cells. **A** Volcano plot illustrating DEPs between the rhein-treated and model groups. Red and blue dots represent significantly upregulated and downregulated proteins, respectively. **B** GO enrichment analysis of the DEPs. **C** KEGG pathway enrichment analysis of DEPs. **D** Heatmap depicting coordinated expression patterns among DEP classes. **E** PPI network analysis of the identified DEPs. **F** Schematic overview summarizing the multidimensional effects of rhein on HSC activation. Created with BioRender.com

To systematically elucidate the anti-fibrotic mechanisms of rhein, label-free quantitative proteomics was applied to characterize the proteomic landscape of TGF-β1-activated LX-2 cells following rhein treatment. Quality control of proteome (Fig. S1A–D), correlation analysis (Fig. S1E), and cluster analysis (Fig. S1F) confirmed the high precision and reproducibility of the datasets. A total of 357 DEPs were identified in the rhein-treated group compared with the model group (Table S6). Key pro-fibrotic markers, including COL5A1, COL1A1, COL6A1, FN1, TNC, and SPARC, were significantly downregulated. In contrast, rhein treatment markedly increased the expression of several metabolic and regulatory proteins, notably ALDH1A3, PSAT1, and TRPV2 (Fig. 6A). Functional enrichment analysis indicated that these DEPs were predominantly involved in epithelial and vascular development, metabolism of oxygen-containing compounds, and the organization of ECM and mitochondrial components (Fig. 6B). KEGG pathway analysis further revealed significant enrichment in ECM-receptor interaction, PI3K-Akt signaling, PPAR signaling, and retinol metabolism (Fig. 6C). Heatmap visualization demonstrated coordinated alterations in proteins associated with ECM remodeling, cytoskeletal dynamics, metabolism, and transcriptional regulation (Fig. 6D). PPI network analysis revealed strong functional connectivity among these pathway-specific clusters (Fig. 6E). Collectively, these findings indicate that rhein inhibits HSC activation by suppressing fibrotic signaling and ECM interactions while concurrently modulating metabolic reprogramming, transcriptional regulation, and mitochondrial homeostasis (Fig. 6F).

### Rhein upregulates ALDH1A3 and restores the ATRA metabolic axis

**Fig. 7:**
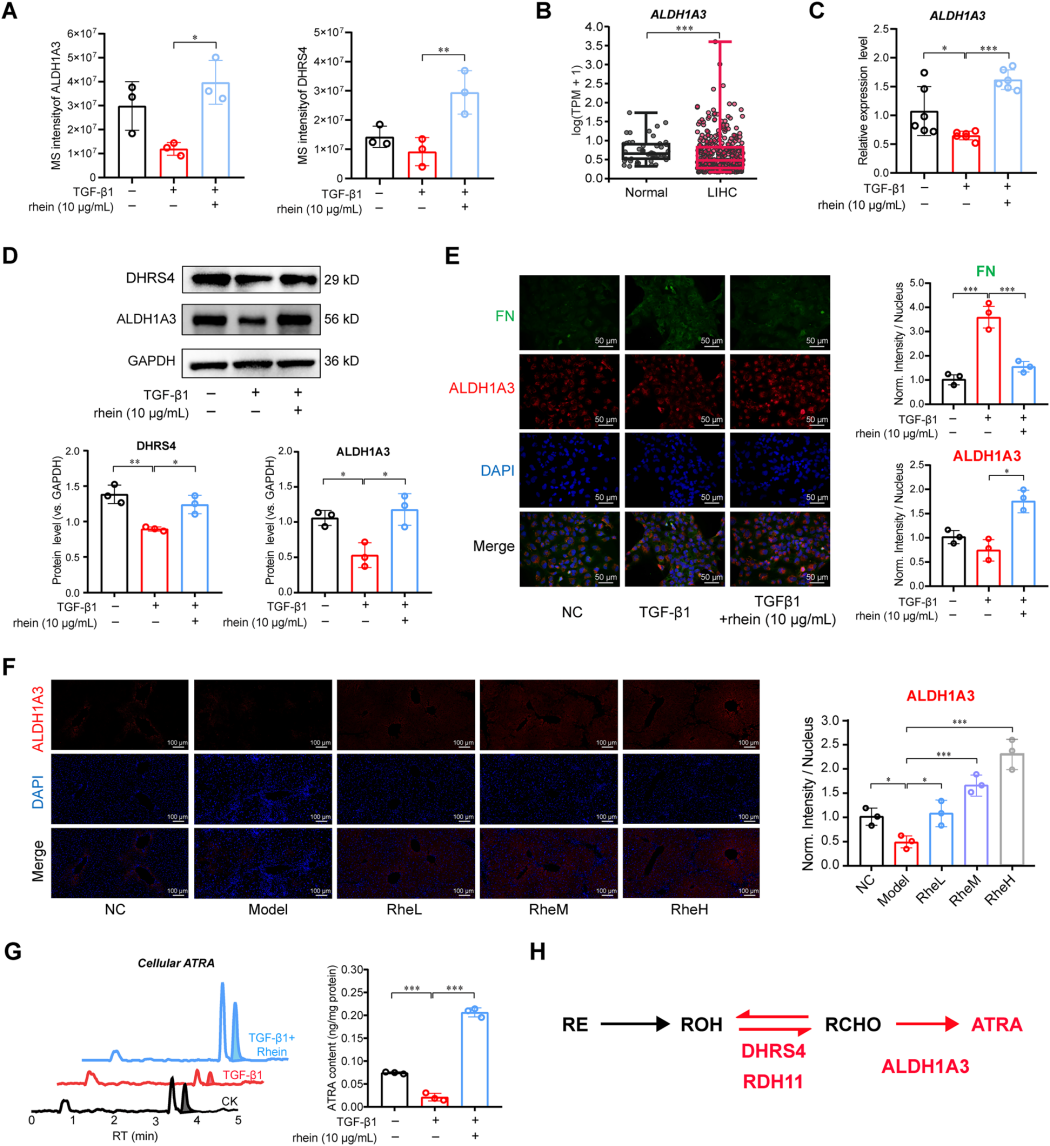
Rhein upregulates ALDH1A3 and restores intracellular ATRA levels. **A** Reduced ALDH1A3 expression in liver LIHC tissues compared with normal liver tissues. **B** Relative protein abundance of ALDH1A3 determined by quantitative proteomics. **C** qRT-PCR analysis of ALDH1A3 mRNA expression. **D** Representative WB images and densitometric quantification of ALDH1A3 and DHRS4 protein expression. **E** Representative IF images and quantification of the normalized fluorescence intensity of ALDH1A3 in LX-2 cells. **F** Representative IF images and quantification of the normalized fluorescence intensity of ALDH1A3 in mouse liver tissues. **G** MRM detection (left) and quantification (right) of intracellular ATRA levels in LX-2 cells. Black, red, and blue indicate the CK, TGF-β1, and TGF-β1 + rhein groups, respectively. **H** Schematic illustration depicting the regulatory effect of rhein on the RA metabolic pathway through the modulation of DHRS4 and ALDH1A3. Data are presented as mean ± SD (n = 3). *p < 0.05, **p < 0.01, ***p < 0.001

To assess the clinical relevance of ALDH1A3 in hepatic pathology, public datasets were interrogated. ALDH1A3 expression was significantly downregulated in liver hepatocellular carcinoma (LIHC) tissues compared with normal liver tissues (Fig. 7A). In TGF-β1-induced LX-2 cells, proteomic profiling revealed a marked decrease in ALDH1A3 abundance, which was significantly reversed following rhein treatment (Fig. 7B). The restorative effect of rhein was further validated at both transcriptional and translational levels. qRT-PCR analysis demonstrated that rhein effectively rescued the TGF-β1-induced suppression of *ALDH1A3* mRNA expression (Fig. 7C). Consistently, WB analysis confirmed a significant upregulation of ALDH1A3 and DHRS4 levels after rhein treatment (Fig. 7D). Likewise, IF staining showed that the reduced ALDH1A3 signal in TGF-β1-treated cells was markedly enhanced by rhein and exhibited a strong inverse correlation with FN deposition (Fig. 7E). Consistent with these in vitro findings, analysis of mouse liver tissues demonstrated that rhein administration effectively rescued the TGF-β1-induced reduction of ALDH1A3 in a dose-dependent manner (Fig. 7F). Given that ALDH1A3 is a rate-limiting enzyme in RA biosynthesis, we quantified intracellular ATRA levels were quantified using a targeted LC–MS/MS approach. TGF-β1 stimulation significantly decreased ATRA levels, whereas rhein treatment effectively restored intracellular ATRA concentrations (Fig. 7G). Collectively, these findings indicate that rhein inhibits HSC activation by upregulating ALDH1A3 and subsequently restoring the RA metabolic pathway (Fig. 7H).

### Rhein directly interacts with ALDH1A3

**Fig. 8:**
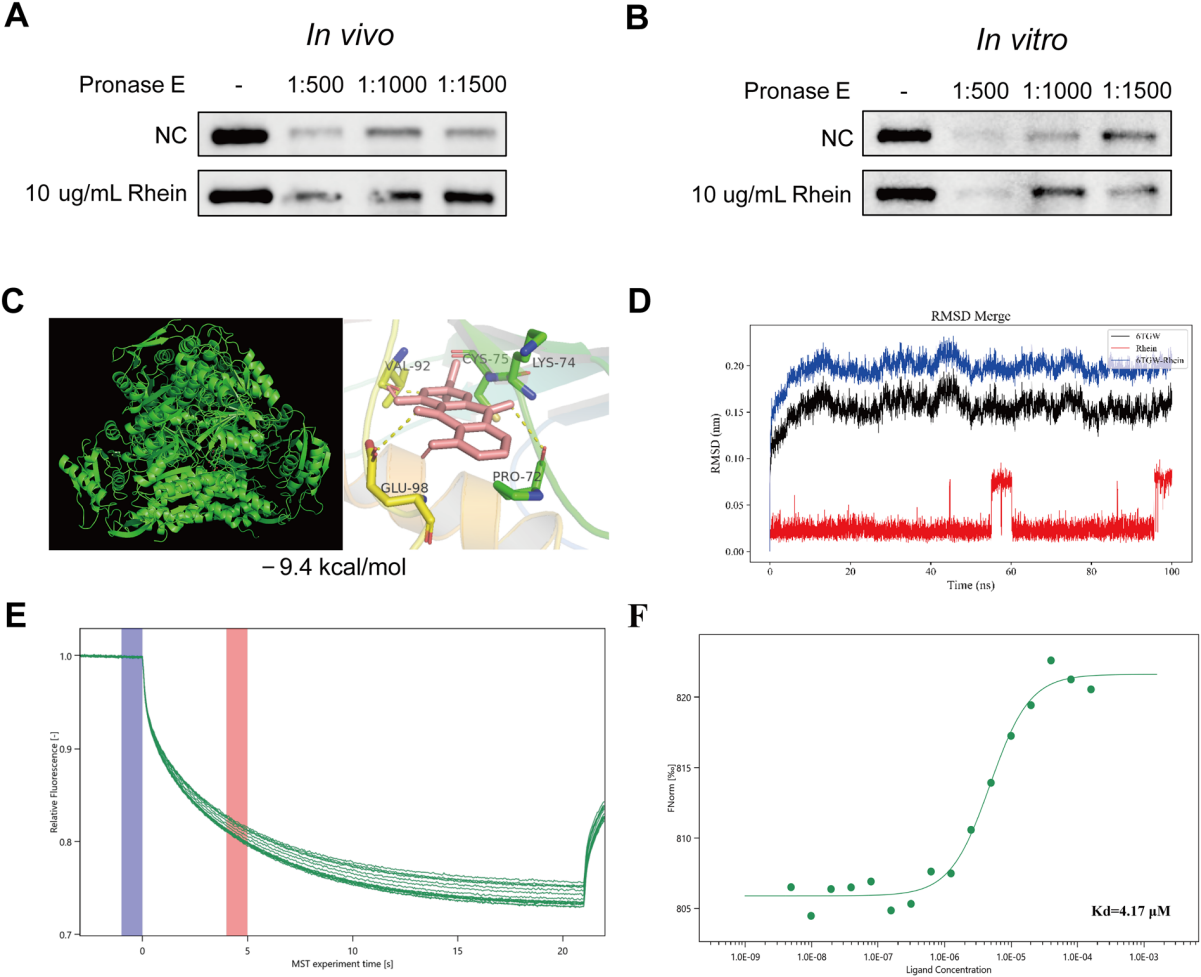
Rhein directly targeted ALDH1A3 protein. **A** In vivo DARTS assay demonstrating the resistance of ALDH1A3 to pronase E digestion in the presence or absence of rhein. **B** In vitro DARTS assay showing increased protease resistance of ALDH1A3 following rhein treatment. **C** Molecular docking analysis illustrating the predicted binding mode of rhein to ALDH1A3, with a binding energy of − 9.4 kcal/mol. Key interacting residues are indicated. **D** Molecular dynamics simulation presenting RMSD trajectories of free ALDH1A3 and the rhein-ALDH1A3 complex over a 100 ns simulation period. **E** MST raw fluorescence traces of the ALDH1A3 titrated with rhein. **F** MST binding curve showing rhein binds ALDH1A3 with a Kd of 4.17 μM

To determine whether rhein directly interacts with ALDH1A3, DARTS assays were conducted. Rhein treatment, both in vitro (cell lysates) and in vivo (cell culture), significantly increased the proteolytic resistance of ALDH1A3 (Fig. 8A, B). Molecular docking analysis was performed to characterize the binding mode at the structural level. Rhein was predicted to occupy the catalytic pocket of ALDH1A3 with high binding affinity (binding energy: − 9.4 kcal/mol). The interaction was stabilized by a network of hydrogen bonds and hydrophobic interactions with key residues, including Pro72, Lys74, Cys75, Val92, and Glu98 (Fig. 8C). MD simulation further confirmed the conformational stability of the ALDH1A3-rhein complex (Fig. 8D). MST was applied to characterize the interaction between rhein and ALDH1A3. Real-time fluorescence traces clearly captured the thermophoretic behavior of the labeled protein across a gradient of rhein concentrations (Fig. 8E). Subsequent quantitative fitting of the fluorescence signals to a dose–response curve demonstrated that rhein bound to ALDH1A3 with a dissociation constant Kd of 4.17 μM, confirming their direct molecular interaction (Fig. 8F).

### Inhibition of ALDH1A3 partially attenuates the anti-fibrotic effects of rhein

**Fig. 9:**
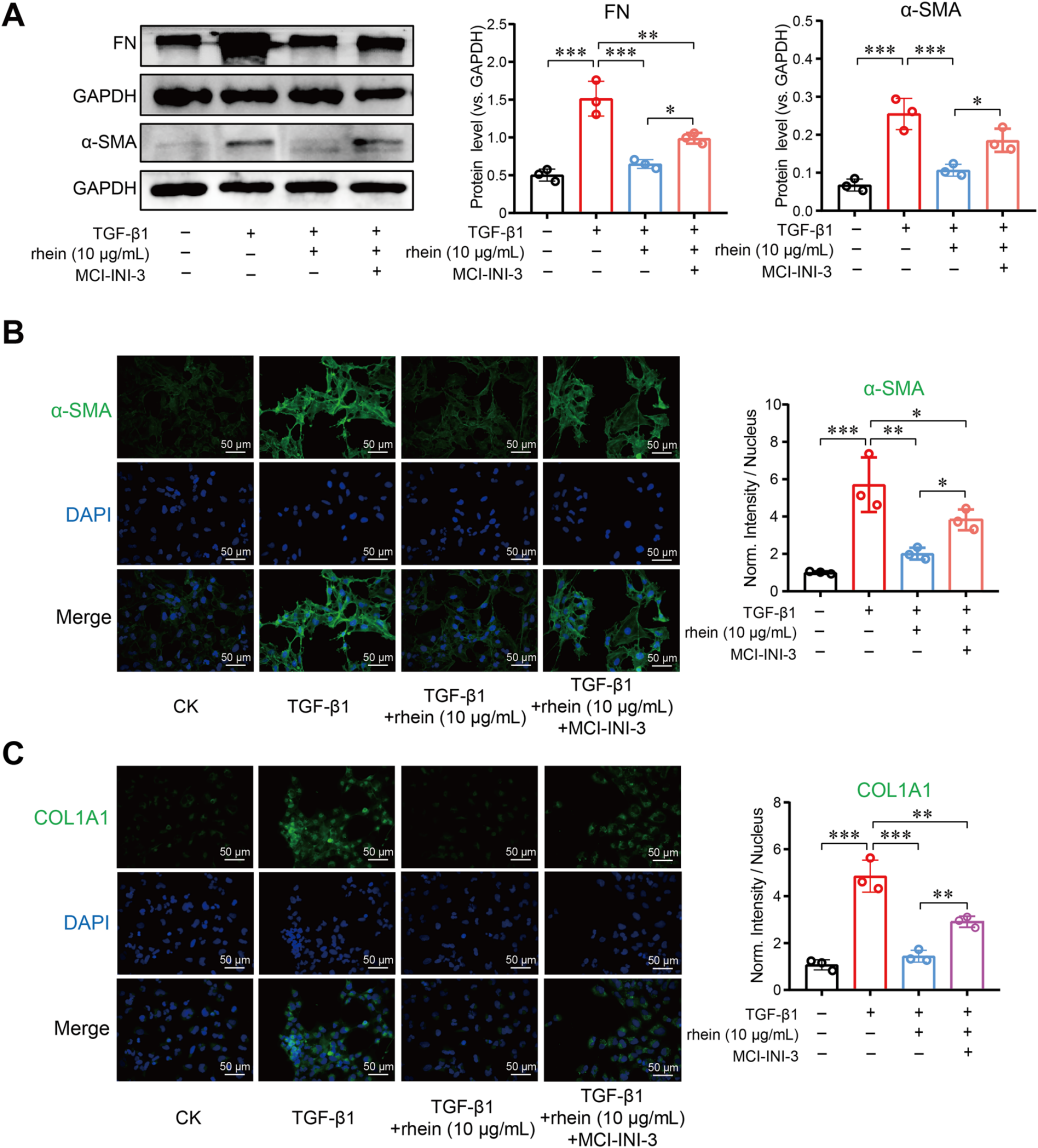
Inhibition of ALDH1A3 by MCI-INI3 abrogates the anti-fibrotic effects of rhein in LX-2 cells. **A** Representative WB images and densitometric quantification of FN and α-SMA protein expression. **B**, **C** Representative IF images with quantitative analysis of α-SMA (**B**) and COL1A1 (**C**) expression. Data are presented as mean ± SD (n = 3). *p < 0.05, **p < 0.01, ***p < 0.001

To determine whether the anti-fibrotic effects of rhein are mediated by ALDH1A3, the ALDH1 inhibitor MCI-INI-3 was employed. The inhibitory effect of the inhibitor on ALDH1A3 were examined (Fig. S2). qPCR and WB analyses demonstrated that rhein (10 μg/mL) significantly suppressed the TGF-β1-induced expression of FN and α-SMA (Fig. 9A). However, this inhibitory effect was partially abrogated by co-treatment with MCI-INI-3. IF staining of α-SMA and COL1A1 further confirmed that the rhein-mediated reduction in ECM deposition mediated was counteracted by ALDH1A3 inhibition (Fig. 9B, C).

### ALDH1A3 mediates anti-fibrotic efficacy of rhein by negatively regulating HSC activation

**Fig. 10:**
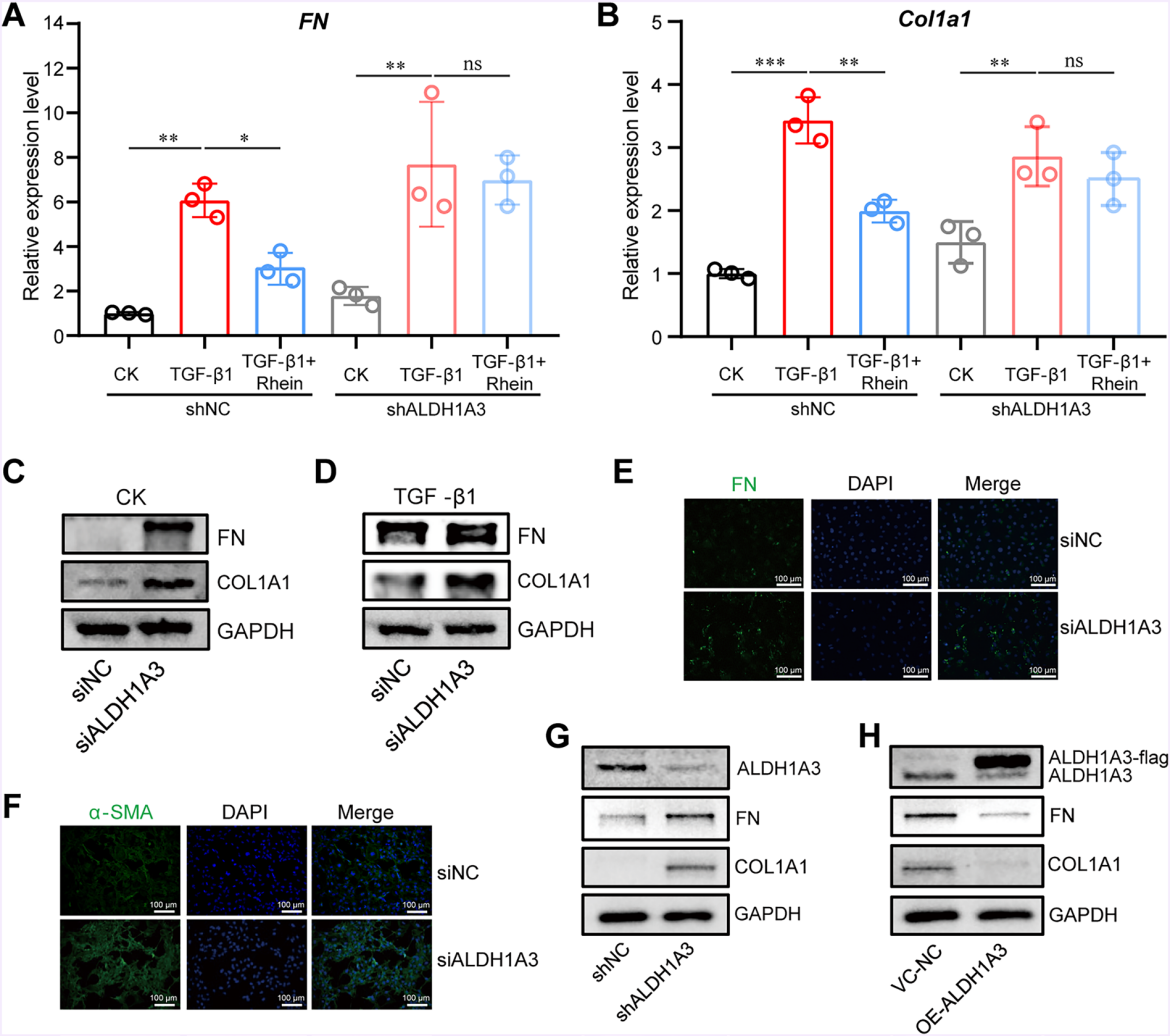
ALDH1A3 serves as a critical negative regulator of HSC activation. **A**, **B** qPCR analysis of *FN* and *Cola1* expression in shNC (**A**) and shALDH1A3 (**B**) cell lines following treatment of TGF-β1 and rhein. **C**, **D** Western blot analysis of FN and COL1A1 expression in LX-2 cells following transient transfection with siALDH1A3 or siNC under quiescent (**C**) or TGF-β1-induced (**D**) conditions. **E**, **F** Representative immunofluorescence images showing the effect of ALDH1A3 silencing on FN (**E**) and α-SMA (**F**) expression in activated LX-2 cells. **G** WB validation of stable ALDH1A3 knockdown (shALDH1A3) and its impact on the protein levels of FN and COL1A1. **H** WB analysis demonstrating that lentiviral-mediated overexpression of ALDH1A3 (OE-ALDH1A3) significantly suppresses the expression of FN and COL1A1 in LX-2 cells. *p < 0.05, **p < 0.01, ***p < 0.001

To further elucidate the role of ALDH1A3, loss-of-function studies were conducted (Fig. S3). Firstly, we evaluated the impact of *ALDH1A3* knockdown on rhein-induced therapeutic effects. PCR analysis revealed that while rhein treatment effectively suppressed TGF-β1-induced mRNA levels of *FN* and *Col1a1* in shNC cells, this suppressive effect was largely abolished in ALDH1A3-silenced (shALDH1A3) cells (Fig. 10A, B). The result suggest that ALDH1A3 is essential for the anti-fibrotic activity of rhein, implying that rhein exerts its therapeutic efficacy, at least in part, by modulating ALDH1A3.

Transient silencing of ALDH1A3 using siRNA (siALDH1A3) significantly increased the expression of pro-fibrotic markers, including FN and COL1A1, under both basal and TGF-β1-induced conditions (Fig. 10C, D). Consistently, IF staining demonstrated that ALDH1A3 deficiency result in a marked elevation of FN and α-SMA fluorescence intensity (Fig. 10E, F). The pro-fibrotic effect of ALDH1A3 loss was further confirmed in a stable knockdown model generated using shRNA (shALDH1A3), which markedly increased FN and COL1A1 protein expression (Fig. 10G). Conversely, the impact of ALDH1A3 restoration on HSC activation was evaluated. Lentiviral-mediated overexpression of ALDH1A3 (OE-ALDH1A3) significantly reduced FN and COL1A1 protein abundance in activated LX-2 cells compared with the vector control (Fig. 10H).

## Discussion

In this study, the chemical profile of FHG was systematically characterized using targeted metabolomics in combination with untargeted analysis of serum-absorbed constituents. An HCS platform targeting anti-HSC activation identified rhein as a principal bioactive compound that effectively suppresses HSC activation and mitigates CCl_4_-induced hepatic fibrosis in mice. Subsequent quantitative proteomic analysis demonstrated that rhein not only inhibits ECM -associated proteins and TGF-β signaling but also regulates mitochondrial function and RA metabolism. This integrated strategy establishes a systematic framework for elucidating the material basis and molecular mechanisms underlying complex Chinese herbal formulas.

Rhein is a widely distributed natural anthraquinone with well-documented pharmacological activities in diverse fibrotic and metabolic disorders. The TGF-β1/Smad pathway is the canonical driver of fibrogenesis. Previous studies in rat models of hepatic [19], renal [26, 27], cardiac [28], and pulmonary fibrosis [29] have demonstrated that rhein blocks TGF-β1 signaling and reduces ECM deposition. Although rhein was initially screened using the HSC activation marker FN, its anti-fibrotic effects were systematically validated in both in vivo and in vitro models. Consistent with prior reports, rhein significantly inhibited HSC activation, as evidenced by decreased expression of α-SMA, COL1A1, and FN (Fig. 4D–F), and mitigated CCl_4_-induced hepatic inflammation and ECM accumulation (Fig. 5G–I). Notably, direct suppression of TGF-β1 was not the most prominent proteomic signature in the present dataset. However, rhein markedly downregulated multiple ECM-associated proteins, including TGF-β2, TGFBI, and integrin αV (Table S6). As an important mediator of fibrosis in IPF and NASH, TGF-β2 exhibits activation patterns partially independent of specific integrins [30]. Given that integrin αV is a key activator of latent TGF-β1 in HSCs [31, 32], its downregulation by rhein further supports the inhibitory effect of this compound on the broader TGF-β signaling network.

To date, investigations into the metabolic regulatory effects of rhein have primarily focused on glucose-lipid metabolism and energy homeostasis [33]. A prominent finding of the present proteomic analysis was the identification of a markedly upregulated metabolic module, namely the RA metabolism pathway (Fig. 6C–E). The RA pathway constitutes an essential link between hepatic Vitamin A storage, HSC quiescence, and fibrogenesis [34]. Under physiological conditions, retinol is oxidized to retinal by the RDH family and subsequently converted to ATRA by the ALDH1A family [35]. These results demonstrated that rhein significantly upregulated key enzymes, including DHRS4, and ALDH1A3 (Fig. 7A–F), resulting in increased intracellular ATRA levels in HSCs (Fig. 7G). These findings suggest that rhein regulates the sequential conversion of retinol → retinaldehyde → ATRA, thereby influencing the fibrotic phenotype of HSCs. Exogenous ATRA suppresses TGF-β1-induced HSC activation by inhibiting HSC proliferation, inducing matrix metalloproteinases (e.g., MMP-3, MMP-13), and repressing pro-fibrotic gene transcription [36]. Previous studies have shown that ATRA markedly suppresses the synthesis of several key extracellular matrix proteins in activated HSCs, although it exerts minimal effects on cellular proliferation [37]. ATRA also mitigate TGF-β-induced HSC activation and vitamin A deficiency–induced hepatic fibrosis through downregulation of thioredoxin-interacting protein [38]. Mechanistic investigations further indicate that ATRA regulates HSC phenotype via nuclear receptors RAR (α, β, γ) and PPARβ/δ, which may exert context-dependent and sometimes opposing biological effects [39]. Activation of RAR-β by ATRA can mechanically reprogram HSCs and suppress their activation [40]. Additionally, the RA-responsive receptor RXR plays an important role. Recent evidence indicates that ATRA-activated RXR-α interacts with CaMKKβ to activate AMPKα, thereby inhibiting HSC activation, proliferation, and migration and ultimately attenuating liver fibrosis [8]. Collectively, ATRA functions as an endogenous regulator that suppresses HSC activation and promotes their reversion to a quiescent phenotype. However, its anti-fibrotic mechanisms are complex and warrant further investigation. Evidence regarding the regulation of ATRA by rhein remains limited. To date, only one leukemia study has reported that rhein synergizes with ATRA to induce differentiation of acute promyelocytic leukemia cells and activate apoptotic pathways [41]. Given that ATRA itself directly inhibits HSC activation [42], exogenous ATRA rescue experiments cannot readily distinguish between pathway-specific and non-specific effects. Therefore, we employed direct target binding assays (MST) and endogenous RA measurement. Their potential interaction in hepatic fibrosis remains to be elucidated.

The conversion of retinol to RA is catalyzed by members of the ALDH enzyme family. ALDH1 constitutes a key subfamily within the ALDH superfamily and comprises several isoforms, including ALDH1A1, ALDH1A2, and ALDH1A3 [43]. Among these, ALDH1A3 is a principal rate-limiting enzyme responsible for the oxidation of retinaldehyde to ATRA. Extensive evidence indicates that reduced ALDH1A3 expression is closely associated with hepatic lipid accumulation, elevated oxidative stress, impaired β-oxidation, and progression of liver fibrosis. Diminished ALDH1A3 activity results in RA deficiency, rendering HSCs more susceptible to activation [9, 10, 44, 45]. The pronounced upregulation of ALDH1A3 observed following rhein treatment is consistent with its role in restoring metabolic flux from retinol toward ATRA. Crucially, we found that the anti-fibrotic effect of rhein was largely abolished upon *ALDH1A3* knockdown (Fig. 10A, B), indicating that ALDH1A3 is indispensable for rhein-mediated suppression of *FN* and *Col1a1*. To comprehensively validate the role of ALDH1A3 in HSC activation, we performed both loss-of-function and gain-of-function experiments. Pharmacological inhibition of ALDH1A3 using MCI-INI-3 substantially attenuated the anti-fibrotic effect of rhein on HSC activation (Fig. 9). Furthermore, siRNA-mediated knockdown of ALDH1A3 significantly increased the expression of pro-fibrotic markers FN and COL1A1 under both basal and TGF-β1-induced conditions (Fig. 10C–G), whereas overexpression of ALDH1A3 markedly reduced these fibrotic markers (Fig. 10H). These bidirectional genetic interventions provide complementary evidence that rhein's anti-fibrotic action is dependent on ALDH1A3 activity. Moreover, stability assays demonstrated that rhein enhanced proteolytic stability of ALDH1A3 (Fig. 8A, B), supporting a direct interaction between rhein and ALDH1A3. Molecular docking analysis further predicted strong binding affinity between rhein and ALDH1A3, involving several key residues (Pro72, Lys74, Cys75, Val92, and Glu98) involved in the interaction (Fig. 8D). To provide biophysical evidence for direct binding, we performed MST assays, which revealed a direct interaction between rhein and recombinant ALDH1A3 protein with a Kd of 4.27 μM (Fig. 8E, F). Collectively, these findings indicate that rhein targets ALDH1A3 and promotes ATRA biosynthesis, thereby restoring the intrinsic anti-fibrotic regulatory capacity of HSCs at the metabolic level.

Although our metabolomics screening identified multiple bioactive compounds in FHG, we focused on rhein as the representative core component due to its abundance and activity, while recognizing that other constituents may contribute to the overall anti-fibrotic efficacy through synergistic or antagonistic interactions. Furthermore, as our findings are based solely on the CCl_4_-induced fibrosis model and given the technical challenges of in vivo HSC-selective ALDH1A3 manipulation, validation in additional fibrosis models (e.g., BDL, NASH) [46, 47] and future studies employing HSC-specific genetic approaches are necessary to establish generalizability and definitive in vivo causality. Finally, the upstream regulation of ALDH1A3 by rhein and the downstream transcriptional consequences of RA restoration remain important directions for future research.

In conjunction with previous reports, these findings indicate that rhein suppresses hepatic fibrosis not only through conventional anti-inflammatory and anti-TGF-β mechanisms but also, more importantly, by targeting ALDH1A3 and restoring the disrupted retinol-ATRA metabolic axis. This process activates a multi-target anti-fibrotic network that facilitates the reversion of activated HSCs to a quiescent phenotype.

## Conclusion

This study identifies rhein as a principal bioactive constituent of FHG and delineates its role in regulating the ALDH1A3-ATRA metabolic axis. Through targeting and upregulation of ALDH1A3, rhein facilitates metabolic remodeling, enhances mitochondrial function, and suppresses HSC activation. These findings establish a mechanistic basis supporting rhein as a potential therapeutic candidate for hepatic fibrosis and highlight the modulation of retinol metabolism as a promising strategy for anti-fibrotic drug development.

## Supplementary Information

Supplementary material 1: Fig. S1. Quality control and identification overview of proteomics data. (A) Mass error distribution of peptides. (B) Peptide length distribution. (C) Summary of basic mass spectrometry identification statistics. (D) Distribution of missed cleavages. (E) Pearson correlation heatmap among samples. (F) Hierarchical clustering heatmap of global protein expression.

Supplementary material 2: Fig. S2. Relative expression of ALDH1A3 under different concentrations of MCI-INI-3 treatment. Data are presented as mean±SD. ***p < 0.001 compared with the CK group.

Supplementary material 3: Fig. S3. Knockdown efficiency of ALDH1A3 siRNAs. (A) qPCR analysis of ALDH1A3 mRNA expression normalized to siNC. Data are mean±SD. ***p < 0.001. (B) Representative Western blot of ALDH1A3 protein levels.

Supplementary material 4: Table S1. Details of natural active compounds.

Supplementary material 5: Table S2. Details of antibodies.

Supplementary material 6: Table S3. Details of primers for qRT-PCR.

Supplementary material 7: Table S4. Details of siRNA and shRNA used for cell transfection.

Supplementary material 8: Table S5. Compounds identified in FHG and seven botanical constituents.

Supplementary material 9: Table S6. DEPs identified using label-free proteomics.

## Data Availability

The mass spectrometry proteomics data have been deposited to the ProteomeXchange Consortium (https://proteomecentral.proteomexchange.org) via the iProX partner repository [48, 49] with the dataset identifier PXD076237.

## Abbreviations

HSC: Hepatic stellate cell
FHG: Huanggen formula
RA: Retinoic acid
HCS: High-content screening
ATRA: All-trans retinoic acid
ECM: Extracellular matrix
MASLD: Metabolic dysfunction-associated steatotic liver disease
FN: Fibronectin
ALDH1A3: Aldehyde dehydrogenase 1 family member A3
FBS: Fetal bovine serum
PFD: Pirfenidone
NC: Normal control
MRM: Multiple reaction monitoring
IHC: Immunohistochemistry
IF: Immunofluorescence
WB: Western blot
DEPs: Differentially expressed proteins
GO: Gene ontology
CETSA: Cellular thermal shift assay
DARTS: Drug affinity responsive target stability
ANOVA: One-way analysis of variance
HYP: Hydroxyproline
TIC: Total ion chromatograms
XIC: Extracted ion chromatograms
