# Supplementary figures and images for: Rhein from a traditional herbal formula attenuates hepatic fibrosis via ALDH1A3-mediated retinoic acid metabolism
Source: Chin Med. 2026 Jul 14;21:188. doi: 10.1186/s13020-026-01465-2 (PMC13366891; doi:10.1186/s13020-026-01465-2)

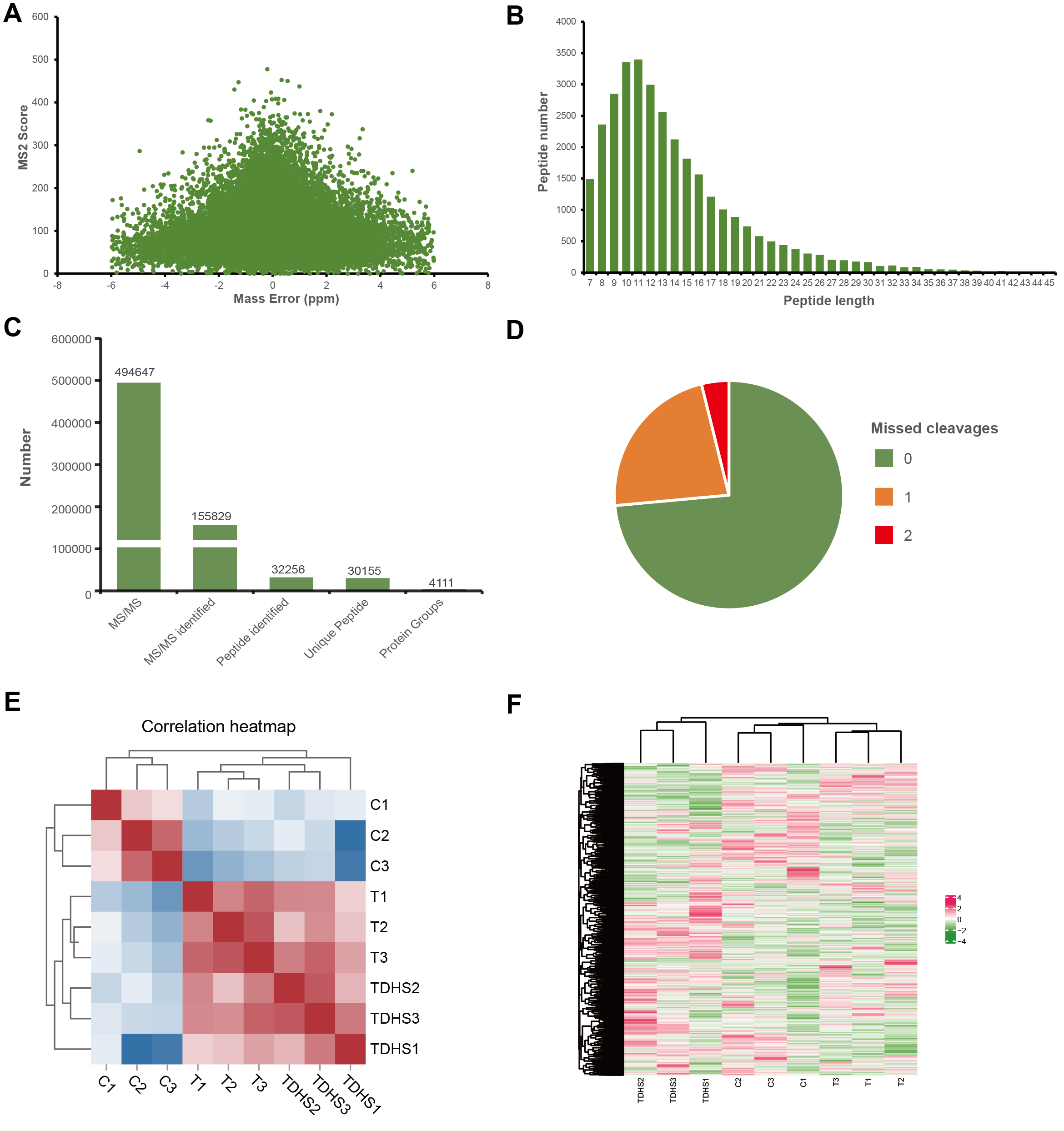

Supplement: Supplementary file 1 — Supplementary material 1: Fig. S1. Quality control and identification overview of proteomics data. (A) Mass error distribution of peptides. (B) Peptide length distribution. (C) Summary of basic mass spectrometry identification statistics. (D) Distribution of missed cleavages. (E) Pearson correlation heatmap among samples. (F) Hierarchical clustering heatmap of global protein expression. [file 13020_2026_1465_MOESM1_ESM.png]

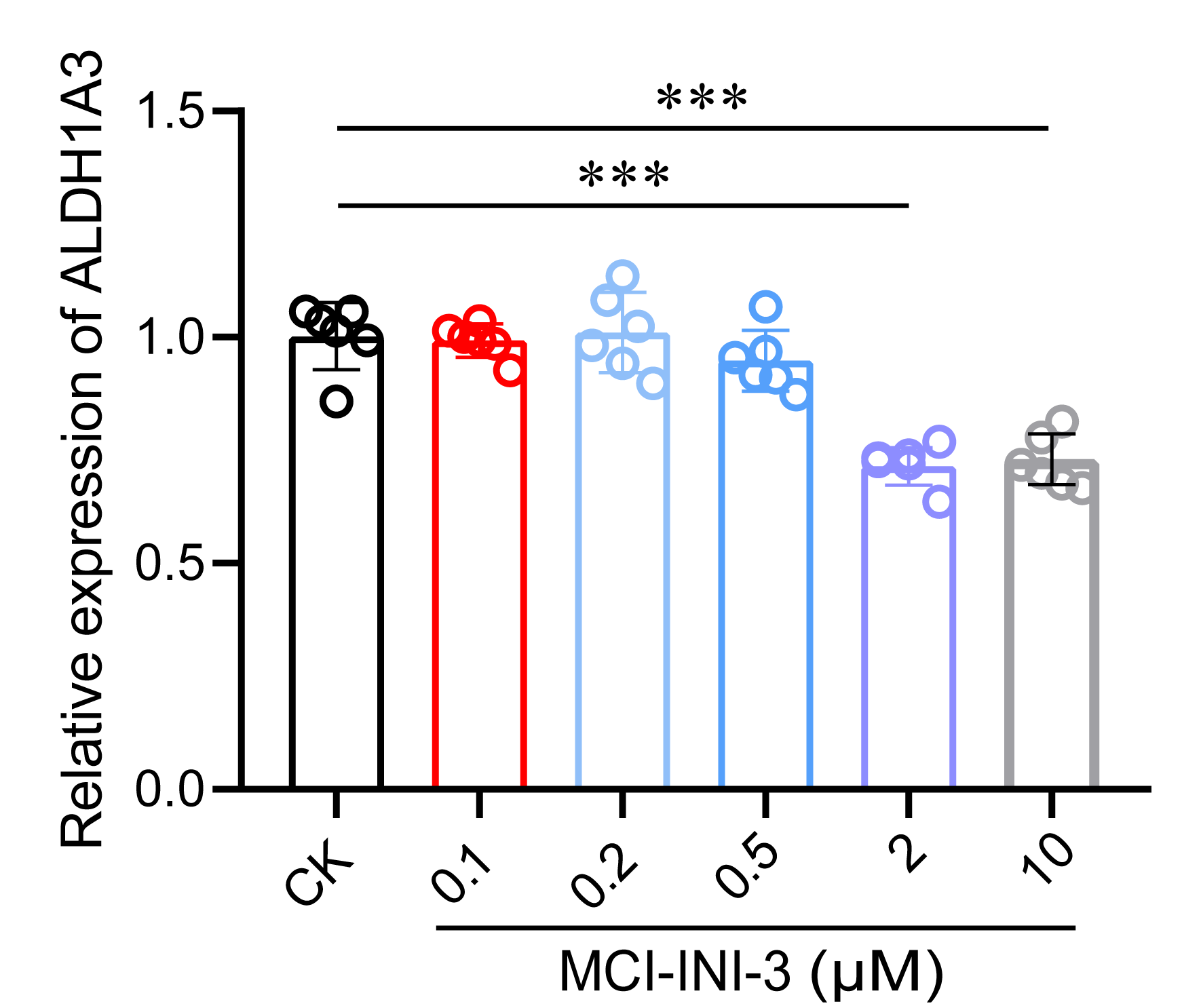

Supplement: Supplementary file 2 — Supplementary material 2: Fig. S2. Relative expression of ALDH1A3 under different concentrations of MCI-INI-3 treatment. Data are presented as mean±SD. ***p < 0.001 compared with the CK group. [file 13020_2026_1465_MOESM2_ESM.png]

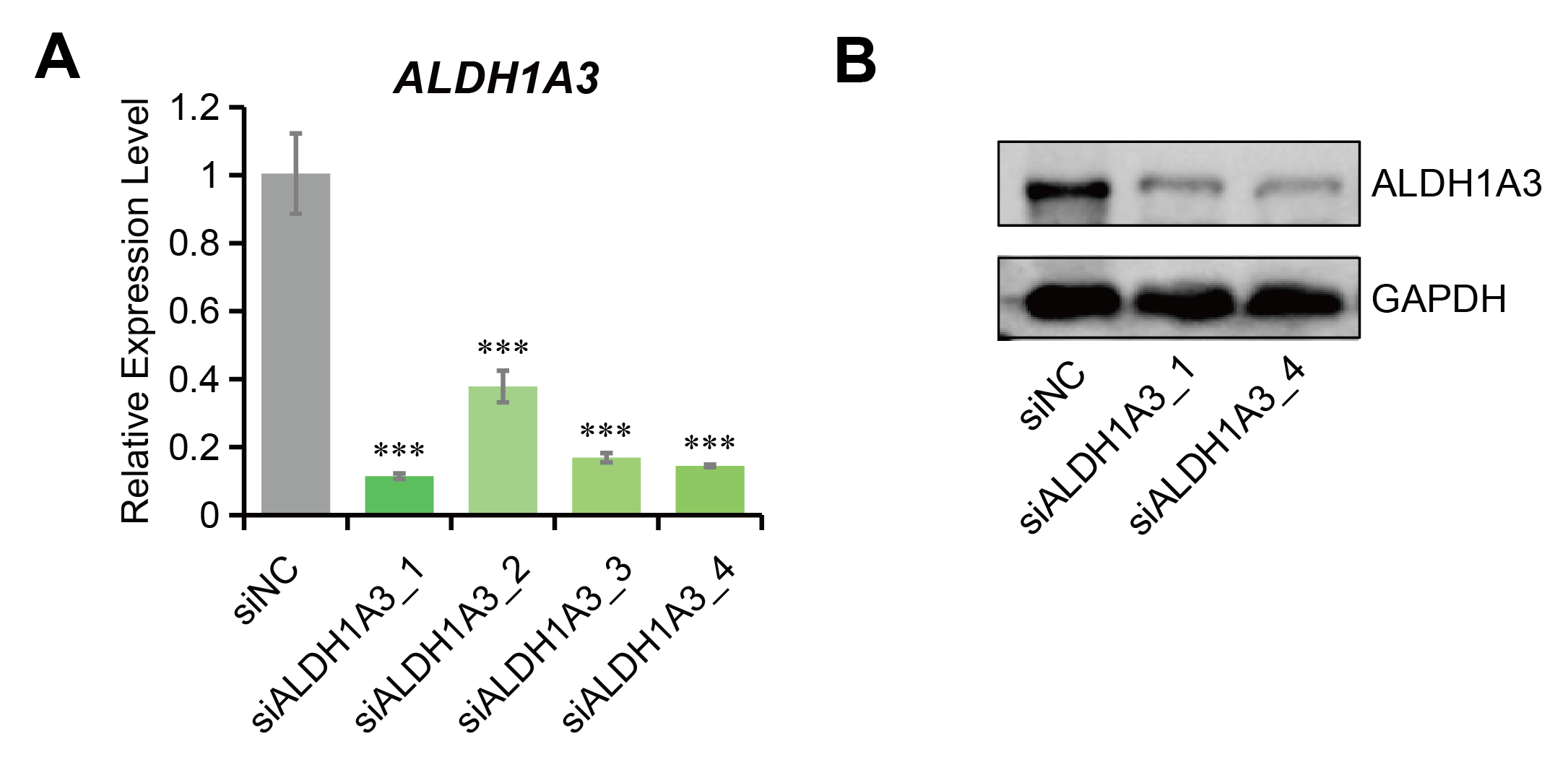

Supplement: Supplementary file 3 — Supplementary material 3: Fig. S3. Knockdown efficiency of ALDH1A3 siRNAs. (A) qPCR analysis of ALDH1A3 mRNA expression normalized to siNC. Data are mean±SD. ***p < 0.001. (B) Representative Western blot of ALDH1A3 protein levels. [file 13020_2026_1465_MOESM3_ESM.png]
